# Congenital, Acquired, and Trauma-Related Risk Factors for Thoracic Outlet Syndrome—Review of the Literature

**DOI:** 10.3390/jcm12216811

**Published:** 2023-10-27

**Authors:** Krystian Maślanka, Nicol Zielinska, Piotr Karauda, Adrian Balcerzak, Georgi Georgiev, Andrzej Borowski, Marek Drobniewski, Łukasz Olewnik

**Affiliations:** 1 Department of Anatomical Dissection and Donation, Medical University of Lodz, 90-419 Lodz, Poland; krystian.maslanka@stud.umed.lodz.pl (K.M.); nicol.zielinska@stud.umed.lodz.pl (N.Z.); piotr.karauda@umed.lodz.pl (P.K.); adrian.balcerzak@stud.umed.lodz.pl (A.B.); 2 Department of Orthopaedics and Traumatology, University Hospital Queen Giovanna—ISUL, Medical University of Sofia, 1527 Sofia, Bulgaria; georgievgp@yahoo.com; 3 Orthopaedics and Paediatric Orthopaedics Department, Medical University of Lodz, 90-419 Lodz, Poland; andrzej.borowski@umed.lodz.pl (A.B.); marek.drobniewski@umed.lodz.pl (M.D.)

**Keywords:** thoracic outlet syndrome, risk factors, congenital, acquired, trauma related, thoracic outlet, anatomical variations

## Abstract

Thoracic outlet syndrome is a group of disorders that affect the upper extremity and neck, resulting in compression of the neurovascular bundle that exits the thoracic outlet. Depending on the type of compressed structure, the arterial, venous, and neurogenic forms of TOS are distinguished. In some populations, e.g., in certain groups of athletes, some sources report incidence rates as high as about 80 cases per 1000 people, while in the general population, it is equal to 2–4 per 1000. Although the pathogenesis of this condition appears relatively simple, there are a very large number of overlapping risk factors that drive such a high incidence in certain risk groups. Undoubtedly, a thorough knowledge of them and their etiology is essential to estimate the risk of TOS or make a quick and accurate diagnosis.

## 1. Introduction

Thoracic outlet syndrome (TOS) is a group of symptoms that result from compression of the neurovascular bundle that exits the thoracic outlet. The thoracic outlet is defined as an anatomical area in the lower neck, extended from the supraclavicular fossa to the axilla, and consists of three main spaces, the interscalene triangle, the costoclavicular, and the subcoracoid space [1]. These confined spaces go through relevant structures, which include the subclavian and axillary arteries, their venous counterparts, and the brachial plexus (BP) [2]. A local compression in this area can cause symptoms such as upper extremity pain, weakness, muscle atrophy, pallor, and paresthesia [3]. 

The classifications of TOS were created based on the type of compressed structures and distinguish neurogenic (nTOS), arterial (aTOS), and venous (vTOS) TOS [4]. The most common form is the neurological variety, which accounts for more than 90% of cases. The definite minorities were the venous forms (3–5%) and arterial (1%) forms. Each of these can be related to traumatic, congenital, and acquired factors, although a degree of trauma is usually seen in a majority of TOS cases [5]. 

A representative example of a congenital case is the presence of cervical ribs or an anomalous first rib (FR). Moreover, due to cases of familial occurrence of TOS, researchers are increasingly linking the occurrence of TOS to the presence of specific forms of particular genes [6,7]. Whiplash injuries and falls make up the vast majority of traumatic cases. Injuries can cause TOS either directly, by damaging structures, or indirectly. Deformation of the bone, scalene muscles, or cervical plexus after trauma can trigger compression of the neurovascular structure in the long term. The acquired causes are usually associated with occupation, sports, and repetitive activity. 

Accurately estimating the incidence of TOS is still a challenge for researchers. One of the reasons for these difficulties may be the lack of universal diagnosis criteria [8]. A recent study conducted on a population of one million estimates the incidence of TOS at about 2.5–4 cases per 100,000 people per year in the general population [9]. Therefore, in some groups of people, compared to the general population, the incidence of TOS may be noticeably higher. For example, a study of 1288 female baseball players (ages 15–17) using the TOS symptom provocation test found symptoms in 32.8% of them [10]. TOS typically affects middle-aged adults, especially women, and symptoms develop more frequently between the ages of 20 and 50 [5,8,11]. The degree of TOS symptoms can be severe. In practice, it is commonly misdiagnosed as entrapment syndrome, muscle pain, or radicular pain. Treatment of this syndrome depends on the pathogenesis behind its appearance. Diagnostic precision is crucial because appropriate treatment for TOS produces great results in a significant number of cases [12].

Since correlations between symptoms and TOS subtype are currently denied in the literature, diagnosis is not easy. However, the authors report some correlations between risk factors for TOS and its type. Most patients with nTOS have neck trauma or repetitive stress at work in the medical history that precedes their symptoms. vTOS, also called Paget-Schroetter disease or effort thrombosis, is frequently preceded by excessive activity of the upper limbs [13]. The arterial form of TOS usually appears spontaneously and is almost always caused by the presence of a cervical or abnormal FR [14]. This type can be easily confirmed or ruled out by X-ray imaging [13].

This paper aims to bring together all the risk factors for thoracic outlet syndrome known to date, pledge them to each other, and consider what makes it such a common condition in various populations.

## 2. Anatomy of the Thoracic Outlet—Figure 1

The structures most commonly involved in TOS were the first rib, clavicle, scalene muscles, and pectoralis minor muscle. Local compression by these structures affects the BP and subclavian vessels at points of anatomical restriction, such as the interscalene triangle, costoclavicular space, and subcoracoid space (Table 1). The interscalene triangle is located above the clavicle and is created anteriorly by the anterior scalene (AS), posteriorly by the middle scalene (MS), and inferiorly by FR. This is the most medially located space of these and contains the trunks of the BP and anteriorly located to it, the subclavian artery [8]. 

The costoclavicular space is located between the other two spaces and constitutes the cervicoaxillary canal between the clavicle and the FR. Its superior boundary is the clavicle, the posteromedially FR, and the posterolateral scapula’s upper border. Through this space, there are BP divisions and a subclavian artery, which, as in the interscalene triangle, runs in front of the BP compartments. This is the most common point of compression of the subclavian arteries [15]. Due to the location of the subclavian muscle, some of its anatomical variants may be involved in causing TOS [16]. 

The last area that forms the thoracic outlet is located below the clavicle, including the minor subcoracoid and pectoralis space [15]. Their posterior limitation is the anterolateral chest wall at the level of the second, third, and fourth ribs. The superior limit is the coracoid process of the scapula, to which the pectoralis minor muscle attaches, constituting the anterior limitation [17]. These anatomical restrictions include cords, axillary artery, and axillary vein. In addition, we must remember that, in this space, the neurovascular bundle can be compressed by the chondrocoracoidal fasciculus or clavipectoral fascia [18].

**Figure 1:**
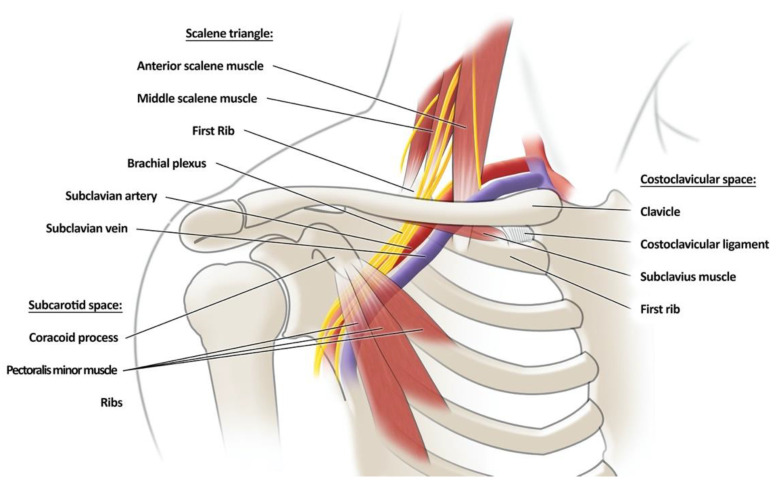
A graphic presentation of the anatomical spaces included in the thoracic outlet complex.

## 3. Congenital Risk Factor

To better illustrate the role of individual risk factors in the induction of TOS symptoms, we can conventionally divide them into risk factors for thoracic outlet compression (CF) and precipitating factors for symptoms (PF). Congenital risk factors overwhelmingly account for CF rather than PF since their occurrence is usually associated with a limitation of the thoracic outlet space, for example, through the presence of an extra rib, additional muscle, or ligaments. To CF, we can also confidently include trauma-related risk factors, which often, through fibrosis, lead to narrowing of the thoracic outlet space. PF, such as repetitive overhead movements, contribute more directly to the onset of symptoms. Paget-Schroetter disease, the situation is a little different. In this syndrome, thrombophilia is a predisposing risk factor, while any risk factor leading to compression or damage to the subclavian vein will be the precipitating factor.

### 3.1. Bone Abnormalities

Variations in the anatomy of the thoracic outlet, both congenital and acquired, are common and mainly include differences in bone and muscle anatomy. Variations in BP anatomy and muscle histology may also contribute. Anatomical causes of TOS can be divided into soft tissue and bone. Soft tissue causes are associated with 70% of TOS cases, while bone abnormalities account for the remaining 30% [5]. According to Weber, 78% of TOS cases were congenital [19]. Aignatoaei et al., in their study, confirmed that all of the bone abnormalities listed below are visible on X-ray, with the exception of pseudoarthrosis, which requires an MRI scan [20].

#### 3.1.1. Cervical Ribs (CR)

Henry et al., in 2018, published a meta-analysis of 141 studies describing correlations between CR and TOS [14]. The incidence of cervical ribs is estimated to be 1.1% in the general population (the vast majority of them remain asymptomatic) and 29.5% in patients with TOS [14]. More than half of them occurred unilaterally. CR is more common among women (70%) than among men (30%) [13,19]. A total of 69% of TOS caused by bone abnormalities are related to the appearance of CR [19]. Its supernumerary rib causes symptoms by compressing essential structures for the proper functioning of the upper limb, such as the BP and subclavian vessels [21]. Henry et al., in their meta-analysis, depict that patients with cervical ribs had more often vascular TOS (51.3%) than neurogenic TOS (48.7%) [14]. The appearance of CR is the most common cause of arterial TOS (especially in patients younger than 40 years), causing arterial emboli, which is responsible for the onset of symptoms [13,21]. Based on the study by Sanders et al., CR is considered a risk factor for nTOS rather than the direct cause because another determiner is needed for the appearance of symptoms [22]. 

There have been several types of cervical ribs described so far. Gruber created their classification in 1869 (Table 2), which remains in use today [23]. In the available literature, there is no information on which types carry a higher or lower risk of TOS. However, researchers agree that type IV (the cervical rib is completely fused with the first thoracic rib) is not only a risk factor for TOS but also carries the highest risk of vascular injury after CR or FR resection during surgical treatment of TOS and is associated with poorer operative outcomes [24]. 

In terms of diagnosis, radiographs almost always reveal a CR [13]. Walden et al., in their work, suggested that CT may detect CR better than MRI, but at the same time, they believe that MRI’s advantage over CT is that it simultaneously reveals anatomical structures that may also be causing complaints [25]. Hardy et al. report that MRI has 100% sensitivity and specificity for the diagnosis of cervical ribs [26]. 

#### 3.1.2. Elongated Transverse Process of C7

**Figure 2:**
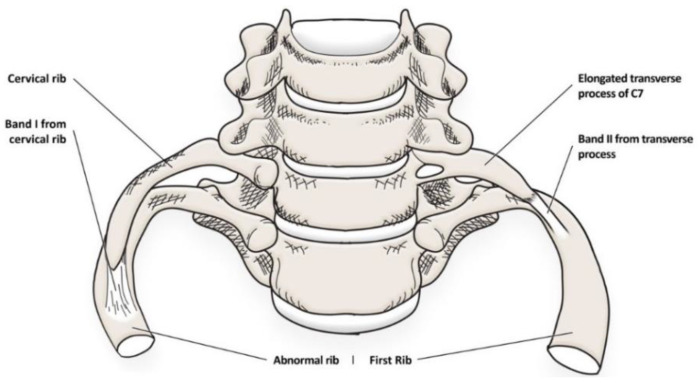
The presentation of congenital fibrous and bony structures that are risk factors for TOS.

The elongated transverse process of C7 may undoubtedly play a similar role in the occurrence of TOS as the cervical rib. Although there are not many reports describing its direct involvement in pathogenesis, its morphology is almost identical compared to that of a cervical rib [27,28]. Obviously, it is much shorter than a cervical rib and can often be narrower. Moreover, the elongated transverse process is in continuity with the C7 vertebra [29]. However, the fact that it is usually shorter (usually 0.5–3 cm) carries a lower risk of this syndrome. However, its occurrence should undoubtedly be noted in this work and should be taken into account as a risk factor—Figure 2.

#### 3.1.3. First Rib Variation

Isolated aberration of the first rib constitutes 9% of all bone abnormalities associated with TOS. Chun-Shin Chang et al. investigated the potential role of FR variation in the pathogenesis of TOS among operative patients [30]. They considered the width, shape, and angulation of FR. They noted that the widths of the sternal end and the vertebral end of FR were considerably wider than in the control group. Based on this, they believe that a wider FR carries a higher risk factor for TOS than a narrower one [30]. 

Abnormalities related to the first rib are the most common cause of venous TOS (may also cause nTOS). Symptoms arise in the pathogenesis of SV compression and thrombosis formation. Congenitally malformed bony tubercle of FR (CMBT) is a common finding during its resection. This kind of tubercle forms a wider and tighter joint at the connection between the sternum and FR [31]. In many cases, a significantly wider tubercle of FR forms the second pseudoarthrosis with the head of the clavicle [32,33]. Researchers noted that a wider and less mobile cost-sternal joint causes immobilization of the medial aspect of the FR and causes the pressure of the CMBT on the subclavian vein, especially while lifting the arm above the shoulder [34]. In the past, CMBT has been confused with hypertrophied costoclavicular ligament and hypertrophied scalenus anticus tubercle [34].

TOS caused by FR abnormalities is usually treated with a first rib resection. This treatment usually relieves symptoms for years, but a recurrence of the condition also occurs [35,36]. When describing the FR variations that cause TOS, we must also mention that after resection, there is a risk of regrowth in the FR that causes the recurrence of the symptoms (although this is rare) [36].

#### 3.1.4. Congenital Pseudoarthrosis of the Clavicle

According to Weber et al., anomalies involving the clavicle account for 22% of the osseous causes of TOS [19]. One of the most common is pseudoarthrosis of the clavicle, but it can also be hypertrophy [37]. In the review presented by Silveira de Figueiredo et al., of the 158 patients with congenital pseudoarthrosis of the clavicle, 42.5% were male and 57.5% female [37]. The right clavicle was affected in 92.6% of cases, 3% in the left clavicle, and 4.4% in both the right and left clavicles [37]. Approximately 80% of these cases were right female clavicles. Bilateral incidence was in the range of 10% and usually cooccurred with other bony aberrations, such as the cervical rib [37]. The TOS caused by this pathogenesis is very rare, but there are some cases described in the literature [38,39,40,41,42,43].

### 3.2. Muscle Variation Associated with Thoracic Outlet Syndrome

In a study by Makhoul et al., out of 200 patients with TOS, 20 cases were associated with the supernumerary scalene muscle. There were 86 cases with scalene developmental variation and 36 cases with subclavius muscle variation [44].

#### 3.2.1. Anterior Scalene Muscle

Collins et al. describe the rare anomaly of absence of the right AS muscle, presenting symptoms and signs of thoracic outlet syndrome [45]. They considered several pathomechanisms that led to the appearance of TOS symptoms in this case. They suspected that the lack of AS was a factor that caused BP to be in proximity to the subclavian vein and artery in a narrowed and abnormal interscalene space or resulted in the neurovascular structures being compressed onto the FR in the costoclavicular space. However, they stressed that the most likely cause of symptoms was the presence of aberrant muscle slips, which would cause compression of the venous and neurological structures [45]. Valera-Calero et al. confirmed in their study that ultrasound imaging is highly reliable for locating and measuring AS morphology [46]. Furthermore, magnetic resonance imaging is also used to study the morphology of AS [47,48,49].

#### 3.2.2. Middle Scalene Muscle

Thomas et al. described MS anatomical variability, which was recognized during TOS operative procedures involving 33 patients [50]. They have recognized anteriorly located (forward on the first rib) insertion of SM in 48% of cases, which further closes an already tight interscalene triangle and causes irritation and entrapment of the muscle located in front of BP. Furthermore, 10% of these patients had extra muscle fibers associated with MS [50]. The literature also describes the accessory middle scalene muscles that caused thoracic outlet syndrome [51].

#### 3.2.3. Scalenus Minimus Muscle

**Figure 3:**
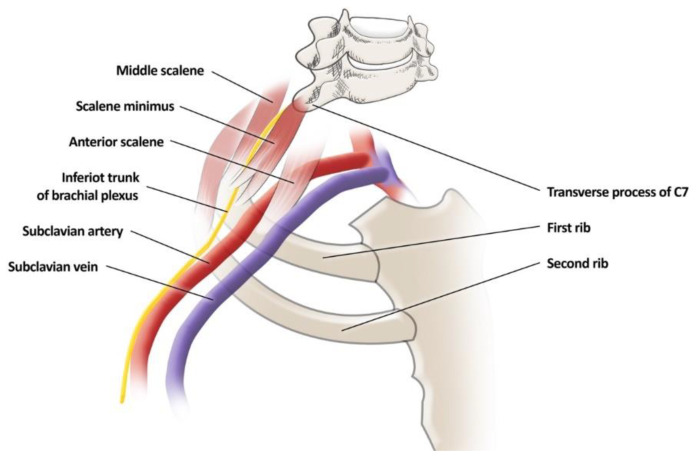
The origin and insertion points of the scalene minimus muscle and position relative to adjacent structures.

SMM is relatively rare among the population. Natsis et al., in their cadaveric study, write about 4.11% of its occurrence, while George et al. note 3% [52,53]. Over the years, many papers have reported a prevalence between 7.8% and even 71.7% [54,55]. Such large differences may be due to the unspecified characteristics of this muscle. It is usually located around MS insertion and has its origin in cervical vertebrae 6 and 7 during insertion on FR. This muscle, as well as additional MS fibers, is most often a cause of entrapment of BP and/or pressure on its structure and/or subclavian vessels—Figure 3 [52,56]. The literature does not describe much in the way of imaging options for the accessory scalene muscles; one paper describes an MRI sensitivity of 50% [26].

#### 3.2.4. Subclavius Posticous Muscle (SPM)—Figure 4

SPM is a supernumerary muscle that is relatively rare. It connects FR to the scapula, and, due to its course over the subclavian vessels, BP might be predisposed toward the development of TOS. Ulusoy et al. screened 350 adults (174 males and 176 females) for its prevalence using magnetic resonance imaging [57]. They detected SPM in 8.3% of the patients (29), with a slight advantage among men (16) over among women (13). A bilateral occurrence of SPM was found in six patients (1.7%). Interestingly, in 60% of these, the SPM was in direct contact with the BP components, while the rest (40%) were not. Akita et al., in their cadaveric study, noted a similar percentage of SPM occurrence (8.9), but the bilateral incidence was less frequent (0.7%) compared to the study mentioned above (1.7%) [58]. For the diagnosis related to SPM, clinicians recommend the use of high-resolution magnetic resonance imaging, with which even its absence can also be recognized [57,59,60].

**Figure 4:**
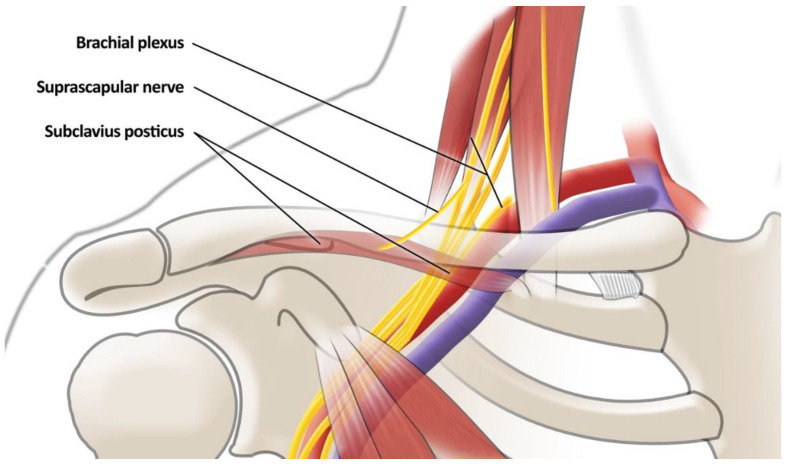
The insertion and origin points of the subclavius posticus muscle and position relative to adjacent structures.

#### 3.2.5. Ectopic Insertion of the Pectoralis Minor (PM) 

Ectopic PM insertion is associated with thoracic outlet syndrome. Asghar et al. performed a meta-analysis that includes 4146 pectoralis minor muscles [61]. Their calculations indicate an insertion of 19.27% prevalence of ectopic PM tendon in the population. Le Double created a classification of the ectopic insertion of the pectoralis minor (Table 3) [62]. Type II appears to be the least favorable due to the fact that it is the only one that crosses the coracoid process (CP) from below, reducing the space under the muscle the most and potentially causing the highest risk of compression of TOS-related structures. Unfortunately, according to this meta-analysis, this is the most common (42%) subtype of ectopic insertion. These anatomical variations can be visualized by ultrasound [63], CT [64], and MRI [65,66].

### 3.3. Brachial Plexus Variation Associated with TOS

The neurological variety makes up the vast majority of TOS types. The compression almost always involves BP structures. The most common anatomical variation associated with the occurrence of nTOS is the pierced anterior scalene muscle by the portions of the BP. Leonhard et al. examined the location of the BPs in relation to AS on 65 cadavers [67]. The classical anatomical course of these structures was present in only 31 of them (47.7%). All the rest of the cases were anatomical variations, which the authors classified as follows:

1. Single-piercing type—the superior trunk of BP passing through AS;
2. Multiple-piercing type—superior and middle trunk of BP separately piercing AS;
3. C5 piercing type;
4. C5 anterior type—C5 and C6 coursed in front of the AS.

The frequency of each type was described by Leonhard et al. in two studies [67,68]. A comparison of their results is shown in Table 4.

Interestingly, Leonhard et al., in their 2016 work, noted that the vast majority of AS punctures occurred unilaterally and almost always on the left side. The authors did not observe any sex differences in frequency [67]. 

In a 2017 paper, the authors identified AS piercing variants as a risk factor for nTOS. In addition, they investigated the possibility of detecting these variants by ultrasound. They concluded that, in combination with patient history and proactive testing, ultrasound is a reliable way of diagnosing this etiology of nTOS. They also believe that the identification of these variants is necessary for developing treatment plans and diagnosing nTOS [68] using magnetic resonance neurography.

### 3.4. Inborn Ligaments and Bands 

Fibromuscular anomalies of the thoracic outlet were first described in 1976 by Roos [69]. He noted the presence of many fibrous and muscular accessory fibers in the area, which he classified into 14 types. Each of these types is presented and briefly described in Table 5. In 2011, Tokat et al. also published a paper describing thoracic outlet anomalies [70]. On the basis of their observations, they wrote that these types of bands may increase the risk of TOS. The occurrence of these variations was observed in 98% of the surgical series, while in the cadaver series, it was observed in 33%. To date, types 3, 1, and 6 have been observed most frequently, particularly in surgical series. In the research described by Tokat et al., types 3, 2, 9, and 12 were the most common, while in the series described by Roos, types 3, 5, 6, and 1 were the most common [69,70]. In view of this, we can confidently hypothesize that type 3 is the most common, but each of the types shown in Table 5 has the potential to limit space and increase the risk of entrapment, contributing to the appearance of TOS. Chavhan et al., in their study conducted among children with TOS, successfully used MRI to visualize inborn ligaments or bands [71].

**Table 4:** Leonhard et al. research comparison.

Type	Leonhard et al., 2016 [71]	Leonhard et al., 2017 [72]
Cadavers examined	65	95
Classic anatomy	47.7%	33.7%
Single piercing	38.5%	52.7%
Multiple piercing	3.1%	4.2%
C5 piercing	3.1%	3.2%
Anterior	3.1%	4.2%

### 3.5. Superior Pleural Sinus 

Hardy et al. described another potential site of neurovascular bundle compression associated with TOS [26]. The space bounded by the ligament originates at C7 or FR and attaches to the suprapleural membrane, labeled the superior pleural sinus. As many as 28% of patients treated surgically for TOS in their research had potential significantly superior pleural sinus identified in the pathogenesis of TOS [26]. In another study, Hardy et al. report that magnetic resonance imaging has a sensitivity of 28% to diagnose this kind of change among patients with TOS [26]. There is little information on imaging techniques for this structure in the available literature.

## 4. Acquired Risk Factors

### 4.1. Changes in Muscle Structures Lead to TOS

#### 4.1.1. Hypertrophy of the Anterior Scalenus Muscle

The first hypothesis on the involvement of AS in the pathogenesis of TOS was formulated in 1935 by Ochsner et al. and concerned the increased tension of AS [1]. This theory was supported by histological examination, which confirmed the predominance of fibers of type 1 (slow twitch) and hypertrophy [53]. There are many research studies describing the occurrence of TOS due to AS hypertrophy [1,72,73,74,75]. This is most often due to various types of exercise. Interestingly, it is not only its large size that can cause this ailment; its absence can also cause TOS, but this is a very rare case [45]. As mentioned in Section 3.2.1, ultrasound imaging is highly reliable for measuring and locating the morphology of AS [46]. MRI is also used to study the morphology of AS [47,48,49]. Hardy et al., in their study, report that MRI has an 81% in diagnosing hypertrophy of the anterior scalene muscles among patients with TOS [26]. 

#### 4.1.2. Hypertrophy of the Subclavius Muscle

Telford et al., during anatomic dissections, noted that abduction or retraction of the shoulder causes compression on the subclavian vein (against the first rib) by the tendon of the subclavius muscle [76]. In addition, a study by several authors noted that the vast majority of patients with Paget-Schroetter (venous TOS) disorders had hypertrophied subclavian muscle structures [77,78,79]. Note that there may also be transient compression of the subclavian vein by the subclavian muscle, causing transient TOS symptoms such as swelling and pain in the upper limb [80,81]. This is called McCleery syndrome. Moreover, in contrast to Paget-Schroetter disorders, in McCleery syndrome, thrombosis is not present [80,81]. These studies provide indirect evidence that the subclavian muscle may also play a direct role in the pathogenesis of TOS, and the combination of hypertrophy of the subclavius muscle with many abduction and retraction movements of the shoulder can compound the risk of TOS. 

### 4.2. Sports Associated with the Risk of TOS Development: Repetitive Overhead Movements

Sports that have been identified in the literature as a risk factor for developing TOS involve much arm movement above the shoulder line and upper arm strength. Most of the research and case reports concern swimmers and baseball pitchers, but TOS was also reported in other sports, such as softball, football, weightlifting, and water polo [82].

Chandra et al. described the surgical treatment of 41 competitive athletes with TOS symptoms [83]. This series of cases involved 13 baseball players, 13 swimmers, five polo water players, four rowers, and two solo athletes. A total of 66% of them were treated for neurologic variants of TOS, while 34% had Paget-Schroetter syndrome (PSS). A total of 85% of the treated athletes returned to full competitive levels. Unfortunately, recurrence of the ailment has also been reported, sequentially 7% in nTOS and 14% in Paget-Schroetter cases [83]. 

Beteck et al. in 2019 published research involving 564 patients subjected to first rib resection and splenectomy (FRRS) for nTOS, and only 33% (184 patients) completed surveys [84]. Among them, 53% (97 patients) were athletes, and 47% (87) were nonathletes. Similar to Chandra et al. publication, most athletes are baseball/softball players; there were as many as 47. The second and most numerous group was volleyball players, 11, 8 gymnasts, and, surprisingly, only 2 (2.06%) swimmers (compared to the Chandra et al. study—35.13%) [83,84]. 

Given these reports, the aforementioned sports can be confidently considered as a risk factor for nTOS, as the authors mentioned [82]. In the literature, we can also find single case reports of the occurrence of nTOS in athletes of other sports (with less overhead motion), such as soccer, cycling, judo, running, climbing, or triathlon, and the authors of these cases agree on the hypothesis that it may be related to the strength training required to prepare in these disciplines [85,86,87,88,89,90]. Beteck et al. have not noted a difference in the effectiveness of treatment between the group of athletes and nonathletes [84]. 

### 4.3. Lower Position of the Shoulder Girdle

Cho et al., in their study, claimed that the position of the shoulder girdle relative to the thorax could affect the incidence of nTOS. This research shows that patients with nTOS (63 X-rays) have a significantly lower position of the shoulder girdle than the control group (126 patients with carpal tunnel syndrome who were matched for age and sex). They used plain cervical anteroposterior and lateral radiographs in which they compared the level of the clavicle (because they expected the position of the clavicle would usually be associated with the position of the entire shoulder girdle) relative to the thorax, using the position of the cervical vertebrae [91].

Statistical results confirmed that patients with nTOS had a lower shoulder girdle position than the control group. The authors confirmed this relationship, further stating that it is a risk factor for nTOS [91].

### 4.4. Hypermobility of the Shoulder Joint

Hypermobility at the shoulder joint has been associated with the presence of TOS. Hudson et al. (1995) published research on this hypermobility and noticed an interesting correlation [92]. A total of 378 patients were studied; hypermobility was detected in 50 of them. Interestingly, 54% of people with hypermobility also had TOS symptoms [92]. 

The main cause of the shoulder joint is ligamentous laxity [93]. If laxity is introduced, the material properties of the ligament can be altered, which can lead to a structural response of the connective tissue. This healing and remodeling can lead to the appearance of connective tissue scars around the subclavian vessels and BP, which can cause symptoms of TOS [94,95,96]. Instability can, of course, also be a direct cause, for example, by lowering the shoulder joint girdle [91]. 

### 4.5. Occupations Associated with a Risk of TOS

Many occupations involve repetitive movements, some of which require the use of the upper limb in the elevation. Repeated movements with the arm raised and carrying heavy objects with the arm outstretched, lifting, or reaching overhead cause increased pressure in the thoracic outlet area. In addition, work in which sedentary posture predominates (computer workers, musicians) can also contribute to symptoms through prolonged cervical flexion or internal rotation of the shoulder, particularly high-performance musicians with a long history of practice, as described by Demaree et al. in their case series [97]. Franklin listed several occupations that carry a risk of nTOS incidence: dental hygienists, shelf stockers, welders, beauticians, drywall hangers or plasterers, and assembly line inspectors [98]. 

The most common work-related TOS is the neurological type [98,99]. In the research conducted on 191 workers (industrial and services) who reported symptoms in the cervicobrachial area, 18% of them had thoracic outlet syndrome. TOS symptoms developed in a significantly higher proportion of women (27%) than in men (11%) [100]. Pascarelli et al. surveyed 485 people with work-related symptoms affecting the upper extremity. A total of 70% of these people were computer users, 28% were musicians. Approximately 70% of the 485 surveyed were diagnosed with nTOS [99]. Hagberg et al. also report that construction workers exposed to vibrations are also at increased risk of developing symptoms of TOS [101]. 

In essence, we can conclude from these studies that any job that requires movements at head height or in which a musical instrument or video display screens are used can potentially increase the risk of TOS, and, therefore, we should take this into account.

### 4.6. Thrombophilia

Thrombophilia has long been linked to an increased risk of PSS, especially among the pediatric population [102,103]. Some sources even recommend routine testing for thrombophilia in people with symptoms of TOS [103]. In the available literature, we can find numerous reports of vTOS associated with thrombophilia caused, for example, by protein C deficiency, elevated lipoprotein (A), and many others [102,103,104,105,106].

The use of oral contraceptives (OC) and ovarian hyperstimulation (OH) is a well-established risk factor for venous thrombosis [107]. This amplifies the risk of vTOS due to hypercoagulability in the venous vessels, which is underpinned by compression of the subclavian vein and associated micro-injuries of the vessel wall.

Some authors have linked the occurrence of symptoms to contraceptive use, especially long-term use of oral contraceptives [108,109,110,111]. In the case described by Cramer et al., the only identified risk factors for Paget-Schroetter were long-term OC use and a recent cycle of ovarian hyperstimulation [109]. This patient was found to have bilateral TOS 7 weeks after starting ovarian hyperstimulation.

As an ideal example, we can use the case report presented by Stricker et al. in which they described young female athletes with Paget-Schroetter syndrome who had all three postulates of the Virchow triad predisposed to thrombosis: (1) stasis caused by constriction by CR; (2) increased coagulability as a result of OC use; and (3) vessel wall injury caused by competitive softball participation [112].

As we mentioned above, OC use is a risk factor for Paget-Schroetter syndrome because it can cause hypercoagulability. We hypothesize that other conditions that can also lead to hypercoagulability can also increase the risk of vTOS [113]. 

## 5. Trauma-Related Thoracic Outlet Syndrome (TR-TOS)

### 5.1. Pathophysiology—Trauma of the Shoulder or Neck

**Figure 5:**
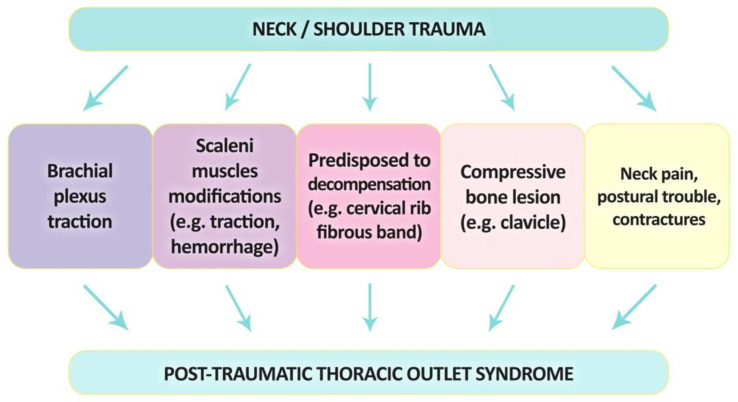
A diagram showing potential pathomechanisms causing trauma-related thoracic outlet syndrome.

Thoracic outlet syndrome typically develops as nerve entrapment syndrome under pathophysiological mechanisms, including a combination of anatomical predisposition and extraneous factors. The predisposing anatomy at the thoracic outlet region is common, which we tried to prove in the previous paragraphs. Extraneous factors leading to decompensation include acquired factors (mentioned above) and neck trauma, as well as shoulder trauma [114]. This type of injury can lead to TOS development in several different ways—Figure 5.

Firstly, BP traction injuries are most often after the extension or acute flection of the neck [115]. This mechanism is responsible for the largest number of nTOS cases. BP can undergo soft, straight traction that causes signs that are immediately noticed by the patient (secondary to whiplash injury) [115,116]. Ide et al. described a series of 119 patients with whiplash injuries caused by traffic accidents. The 45 of 119 (37.8%) injured had nTOS caused by irritation of the BP [116]. Interestingly, more women, compared to men, had a higher BP irritation rate. The left side was injured more frequently than the right. According to this research, driving position is associated with a lower risk of symptoms than among passengers, and fastened seat belts reduce the risk of symptoms [116]. 

Second, BP entrapment may also be caused by fibrotic scar tissue, which is formed in this region as a result of AS and MS traction, microhemorrhages, and injuries to contiguous structures [117]. Several research studies agree that the direct cause of symptoms in this pathophysiological mechanism is the narrowing of the interscalene triangle as a consequence of the modification of the structure of AS and MS after trauma [114,118,119,120]. This is the most described cause of TR-TOS in the literature. Sanders et al. described the changes that occur in the structure of AS and MS after injury occurring in patients with TOS [117]. They also noted a definite predominance of type 1 fibers over type 2 in muscle structures after injury. In comparison, this muscle has an approximate amount of both types of fibers. Additionally, post-traumatic muscles had a significantly increased connective tissue content. These observations were consistent with a history of neck injury [117].

As we mentioned in earlier paragraphs, the thoracic outlet can be narrowed by various structures such as CR, prolonged TP, additional scalene muscles, scars, or inborn ligament or bands. If such a situation occurs, we are talking about a predisposed thoracic outlet, which is also a significant risk factor for TR-TOS. The predisposed thoracic outlet can be decompressed by the sudden force received at this location at the time of injury. Decompression can occur quickly and most often lead to a neurogenic type of TOS, which several authors in the literature [21,117,121,122].

TOS can occur after fractures of the FR, the transverse process of C7, or the clavicle; eight patients with developed TOS in this pathomechanism were described by Dubuisson et al. [122]. In 1973, Mulder described a few patients who had various forms of TOS after trauma to the collarbone, cervical spine, shoulder, or FR [123]. Casbas et al. reported 13 cases of TOS with procured bone lesions, and in their work, they pointed out that these types of cases may account for approximately 5% of all patients with TOS [124]. In this mechanism, damage to BP can first be explained by edema, bleeding, or compression by bone fragments (due to the fact that free bone block may cause a reduction in the space in the costoclavicular area).

Several authors have hypothesized that spasms or pain of the neck/shoulder muscle (which are often seen after whiplash injuries), as well as poor shoulder posture, can contribute to the development of TR-TOS. It is worth noting that not all individuals who experience a car accident develop TR-TOS, and its occurrence may be influenced by factors such as anatomical conformation, work habits, and pretraumatic posture. The prolonged anticontraction following whiplash injury can result in connective tissue changes and the formation of micro adhesions, which may lead to the development of TR-TOS. 

### 5.2. Incidence of TR-TOS

Trauma-related thoracic outlet syndrome accounts for a very large proportion of the causes of this condition. Ellison et al. wrote about the frequency reaching 49% (single trauma leading to TOS) [114,122]. Among TR-TOS, the most common reason for its occurrence was traffic accidents. In the study described by Dubuisson et al., this reason represented 63% of cases [122]. The force hyperextends the neck and stretches the cranial muscles (most often the scaleni muscles). Muscles stretch, bleed, hypertrophy, and shorten. In the process, the already crowded triangular tunnel through which the neurovascular bundles pass becomes even narrower [122].

In 1978, Woods reported that TOS occurred in 459 of 1958 patients with cervical soft tissue trauma, representing a rate of up to 23% [125]. In a recent article by Ide et al., 45 of 119 patients (37.8%) injured in car accidents were diagnosed with BP irritation [116]. In turn, Ellison et al. reported that TR-TOS among their patients was the most common result of a motorcycle accident (47.78%), followed by falls (20.45%), with traction injury (11.36%) being the third most common reason [114]. These statistics show that TR-TOS is a large percentage of TOS cases. Injuries of this type are not only a direct trigger of TOS but are also a serious risk factor for future symptoms as a long-term complication. The incidence of whiplash injury has increased over the years and is becoming, along with accidents, one of the most common causes of TR-TOS. Acceleration-extension lesions of the cervical spine also account for a large percentage of TR-TOS triggers [122].

### 5.3. TOS Secondary to Inflammations

Weinberg et al. described TOS as a result of arthritis of the first costovertebral joint. In this article, 40 of 232 patients studied (who were tested for TOS) over 6 years of medical practice were found to have this kind of arthritis as a cause [126]. The authors believe that the mechanism that causes this syndrome is that when hydrocortisone is injected into the costovertebral joint, the first intercostal nerve is entrapped as it crosses the narrow space between the first and second costovertebral joints [126].

Another example of inflammation leading to TOS is neuritis of BP. However, in this case, the pathomechanism is completely different from that in the previous paragraph. The author of this paper identified neuritis as the cause of TOS atrophy of the shoulder girdle, leading to biomechanical dysfunction. Muscle weakness leading to an abnormal head or shoulder position has already been described in this study, but the main cause is precisely BP neuritis. The most common nerves in neuritis are the long thoracic, suprascapular, and interosseus nerves [127]. The etiology is often unknown but may occur after surgery or illness [128]. 

## 6. Risk Factors and Treatment

First, the patient must develop symptoms to begin treatment. The cervical rib, for example, occurs in almost 1.1% of the population, of which only a small percentage will develop TOS [25,129]. The choice of treatment strategies depends mainly on the type of TOS present in the patient diagnosed. Treatment of nTOS usually starts with conservative measures, while vTOS, aTOS, and nTOS with refractory symptoms can be treated surgically [130]. 

Conservative strategies include physical therapy (active stretching, targeted muscle strengthening), education (relaxation techniques, posture correction, weight management), pharmacological therapies (NSAIDs, opioids), and injections into the anterior scalenus or pectoralis minor (botulinum toxin type A, steroids, and local anesthetic) [130,131]. Usually, surgery is the next step in nTOS treatment when conservative and physical therapy has failed after 4–6 months, but in vTOS and aTOS, it is very often implemented as the first intervention. The basis of surgical treatment is first rib resection, scalenectomy, scalenotomy, and pectoralis minor tenotomy, which are practiced either alone or in combination [132].

Balderman et al. in 2019 described their observations of the treatment of patients with nTOS. In his research, in which he compared physical therapy with surgery therapy, he did not note any specific pretreatment factors that might indicate a greater effectiveness of physical treatment over surgical treatment and vice versa [12]. The effectiveness of physical therapy was 31%, while the effectiveness of surgical therapy among those who were not helped by physical therapy was close to 90% [12]. Goeteyn et al. came to similar conclusions based on the treatment of patients with nTOS [133]. TOS associated with acquired risk factors may respond better to conservative therapy, especially physical therapy. Working on posture, movement habits, or work ergonomics may have really suitable results [134]. Most acquired risk factors are based on abnormal biomechanics in the shoulder. Levine et al. described in their paper several exercises that had very suitable results in the treatment of TOS. Furthermore, conservative measures seem to be the best treatment option for patients with lower positions and hypermobility of the shoulder joint [93]. Warrick et al., in their study, found that surgical and non-surgical treatment had positive results in the treatment of nTOS in athletes who perform repetitive overhead movements [135]. Beteck et al. compared the results of nTOS treatment between athletes and nonathletes. In their study, athletes had better results with conservative treatment than nonathletes, while no differences were observed between these groups after surgical treatment [84]. 

Undoubtedly, more comparative studies are needed based on the different etiology of TOS to improve the process of selecting therapies and predicting the prognosis of a specific treatment.

## 7. Conclusions

After analyzing and juxtaposing the risk factors for TOS, we should not be surprised that some populations are much more likely to develop symptoms than others. Overlapping factors dramatically increase the risk of developing symptoms. Therefore, detailed knowledge of each is extremely important for assessing the risk and optimizing the diagnostics of TOS. A summary of the risk factors listed in the text is shown in Table 6.

## Figures and Tables

**Table 1:** Crucial anatomical spaces for the pathogenesis of TOS.

Space	Borders	Contents
Scalene triangle	Anterior: Scalenus anterior Posterior: Scalenus middle Inferior: 1st rib	Brachial plexus (trunks) Subclavian artery
Costoclavicular space	Anterior: Clavicle (or subclavicular muscle) Posterolateral: Upper board of the scapula Posteromedial: 1st rib	Brachial plexus (division) Subclavian vessels
Subcoracoid space	Anterior: Pectoralis minor muscle Superior: Coracoid process of the scapula Posterior: 2nd–4th ribs	Brachial plexus (cords) Axillary vessels

**Table 2:** Cervical ribs classification proposed by Gruber [23].

Type	CR Type Description
I	CR extends to the transverse process of the 7th cervical vertebrae
II	CR extends beyond TPs with no connection to FR
III	CR extends beyond TP with partial fusion to FR by fibrous bands or cartilage
IV	CR is fully fused to FR

CR—cervical rib, TP—transverse process, FR—first rib.

**Table 3:** Course of PM relative to the coracoid process, based on the classification of ectopic insertion of PM by Le Double [62].

Type	Type Description (in Relation to Coracoid Process)
I	Only the tendon of PM crosses above CP
II	Tendon of PM crosses above CP, and much of the muscle mass runs under CP
III	The whole PM runs above CP

**Table 5:** Bands and ligaments. Types distinguished by Roos [69].

Type 1 A band from the anterior apex of an incomplete CR to the middle of the upper surface (posterior to scalene tubercle) of the FR. Type 2 A strip originated at elongated C7 that attached to the FR in the same place as a type 1 Type 3 A band that had origin near the neck of FR and insertion behind the scalene tubercle Type 4 A band lies along the anterior edge of SM, which has an origin on the transverse process and is inserted into the FR with SM Type 5 The scalene minimus muscle (SMM)—described in more detail later in this paper Type 6 SMM inserts into Sibson’s fascia (not to the FR) above the cupola of the pleura Type 7 A fibrous strand running in front of AS, down to FR, and inserts to the sternum or costochondral junction (CJ). Due to the close relation to the subclavian vein (right behind it), it may cause her obstruction. Type 8 A strip arising from MS passes below the subclavian vessels (vein and artery) to insert into CJ Type 9 The network filling the inner, posterior arch of the FR, consisting of muscles and their fascias Type 10 AS-derived muscle fibers attaching to the brachial bundle (perinerium) Type 11 Muscle fibers that form bands that connect the AS and AM and run between the cords of BP Type 12 An anomalous AS (inferior part) runs posterior to the C5 and C6 roots Type 13 n band consisting of fused scalene muscles Type 14 Fibrous bands run vertically, behind the AS, and anteriorly to the roots of BP

**Table 6:** Summary table of the risk factors mentioned in the text.

Congenital Risk Factors	Acquired Risk Factors	Trauma-Related Risk Factors
Cervical rib [22] Pseudoarthrosis of the clavicle [38] First rib variation (increased width, congenital malformation) [30] Ectopic insertion of pectoralis minor [62] Morphological variations of scalene muscles (AS, MS, SMM) [45,50] Subclavius posticous muscle presence [58] Brachial plexus variation (piercing AS and/or MS) [67] Superior pleural sinus presence [26] Congenital ligaments and bands [69] Elongated transverse process of C7 [27]	Hypertrophy of the AS, MS, and subclavius muscle [1,72,76] Repetitive overhead shoulder movements [82] Sports associated with risk of TOS (baseball, swimming, water polo, judo, climbing) [82,83] Lower position of the shoulder girdle [91] Hypermobility of the shoulder joint [92] Occupations with overhead movements or sedentary work patterns (e.g., computer users or musicians) [98,99,100]	Whiplash injury car/motorcycle accident [115] Arthritis of the first costovertebral joint and hydrocortisone injection into the costovertebral joint [126] Neuritis of the brachial plexus [127]

## Data Availability

Please contact authors for data requests (Łukasz Olewnik—e-mail address: lukasz.olewnik@umed.lodz.pl).
